# The role of Aboriginal leadership in community health programmes

**DOI:** 10.1017/S1463423621000414

**Published:** 2021-10-29

**Authors:** Victoria Stroud, Josie Adams, Doreen Champion, Geraldine Hogarth, Anne Mahony, Ruth Monck, Trulie Pinnegar, Naomi Sprigg dos Santos, Charles Watson

**Affiliations:** 1 Telethon Kids Institute; University of Western Australia, Crawley, WA, Australia; 2 WA Country Health Service Goldfields, WA, Australia; 3 Curtin University, Bentley, WA, Australia

## Abstract

This is an account of the crucial role played by a strong local Aboriginal workforce in health care delivery. We report on the personal experience of dedicated Aboriginal health professionals across Western Australia. Their understanding of what has worked in the provision of primary health care in their communities emphasises the importance of strong, local collaboration in the development of effective prevention programmes at a community level.

## Introduction

There remains a glaring disparity between the health of an Australian Aboriginal child when compared with that of a non-Aboriginal Australian child (Fogarty *et al.*, 2018a; Fogarty *et al.*, 2018b). In recent years, studies have advocated for the adoption of culturally sensitive health care provision if significant improvements are to be made in the health of Australian Aboriginal children. (Coffin, 2007; Saha *et al.*, 2008; McCalman *et al.*, 2012; Horvat *et al.*, 2014; Bainbridge *et al.*, 2015). These studies emphasise that organisations and providers which have strong culturally sensitive leadership have more effective health outcomes for Aboriginal people (Keatinge *et al.*, 2007; Choi & Kim, 2018; Munns *et al.*, 2018). Equally, the role of effective primary prevention should be enhanced if we are to address diseases of childhood.

This paper argues that culturally appropriate Primary Health Care (PHC) can improve health and wellbeing outcomes for Australian Aboriginal people (Davy *et al.*, 2016). Our own work has found that effective prevention programmes require a collaborative approach and Aboriginal leadership that enables community ownership to ensure that cultural values and practices are embedded in the delivery of PHC. We use an example from work with otitis media in regional and urban areas of Western Australia to support this conclusion.

### Two-way learning

Authors that have examined ways to improve outcomes for Aboriginal children have concluded that there is a vital role for two-way learning – the reciprocal sharing of information that builds knowledge and understanding of Aboriginal cultural values, for example, (Malcolm, 1995; Zubrick *et al.*, 2006; Coffin, 2007).

Two-way learning means a truly collaborative approach where both patients and health professionals are learners, becoming empowered by an understanding of each other’s knowledge, perspectives and skills (Wright, 2011). Two-way learning enables greater self-determination between parties because knowledge and balance of power are shared (Wynne-Jones *et al.*, 2016; Harfield *et al.*, 2018). An approach that facilitates two-way learning combined with flexible and accessible service provision and Aboriginal leadership can help ensure effective PHC programme development and implementation (Stroud *et al.*, 2020).

We came together as part of a sub-committee of the WA Child Ear Health Strategy, called the Enhanced Prevention Working Group. This paper represents reflections of our learning and shared knowledge and experience in our respective roles delivering or supporting prevention in health service delivery. Stories from Aboriginal health professionals throughout Western Australia shed light on ways to improve health outcomes for Australian Aboriginal families. They emphasise the importance of strong, local collaboration in the development of effective PHC programmes that can be adapted to suit the local community. We have looked at how these observations relate to the Indigenous PHC service delivery model as described by Stephen Harfield *et al.* (2018) and are being applied to the Aboriginal Community-Controlled sector (Dawson *et al.*, 2020), also see (Gomersall *et al.*, 2017). We have sought to use this understanding as a basis for the design of a PHC programme that targets language development in Aboriginal children.

### WA Child Ear Health Strategy overview and Enhanced Prevention Working Group

The Enhanced Prevention Working Group was formed to advise a State-wide WA Child Ear Health Strategy (the Strategy) Steering Group on recommendations to operationalise the strategic priority of Enhanced Prevention – one of seven priorities to address ear health in children vulnerable to ear disease. The Strategy is focused on Aboriginal children aged 0–5 years. It recognises that for the prevention and treatment of ear disease to improve, agencies and service providers need to avoid duplication, and to adopt a consistent evidence-based approach to service provision (WA Child Ear Health Steering Committee, 2017). The Working Group membership included representatives from Western Australian Government and Non-Government organisations, including community champions and local Aboriginal health professionals who worked in Aboriginal health programmes. The Group explored local solutions that could guide recommendations for a state-wide prevention programme.

The Working Group found that current strategies to prevent ear disease in Aboriginal communities were in many cases limited by a lack of evidence of effectiveness. Anecdotal advice from within Aboriginal communities suggests basic prevention messages such as ‘*Wash your face and hands with water and soap and use your own towel’* and ‘*Eat good food (local, tinned, frozen or fresh)’* and *‘exercise regularly*’ are not emphasised enough and are generally undervalued. They are often not understood by Aboriginal community members and therefore have limited impact. The final recommendations of the Working Group suggest ways to address this deficiency by stressing the importance of leadership by local Aboriginal health and education professionals in community-based prevention programmes (Stroud *et al.*, 2020).

### Identifying characteristics of Indigenous service delivery models

Harfield *et al.* (2018) identified eight key characteristics (i.e. values, principles and components) of effective Indigenous PHC service delivery models. They found that *culture* underpinned all aspects of effective Indigenous PHC service delivery; the factors relating to the emphasis on culture included community participation, continuous quality improvement, a culturally appropriate and skilled workforce, a flexible approach to care, holistic health care, and self-determination and empowerment. The authors pointed out that attention to culture is mostly lacking from the traditional medical model of care used in Australia (Harfield *et al.*, 2018). They argue that programmes that promote culture through prevention strategies and enhancing protective factors are more likely to have more traction than programmes that are based on a medical model which focuses on treatment and rehabilitation.

## Working together

Our team recently published a paper reporting on the findings of the Working Group, focusing on recommendations for key messages to promote good ear health within Aboriginal communities (Stroud *et al.*, 2020). After realising the central significance of the collaborative approach in the continued learning and professional development of both Aboriginal and non-Aboriginal members, we agreed to prepare a further paper based on these learnings that advocates for a greater role for Aboriginal leadership in PHC.

The Group, therefore, came together via online meeting forum to discuss the approach for this article. Online forums were used due to social distancing requirements and to accommodate authors from regional and remote WA. Authors from the Goldfields were invited to share their personal reflections about what has worked/not worked in managing and enabling an Aboriginal workforce. These authors (AM and NSS) provided an evidence-based outline of their approach and experience of what has worked in their respective roles. The authors who are senior Aboriginal Health Workers also shared their stories and experiences of how they have been enabled, what has worked and what has not worked.

The first author also conducted a preliminary review of literature on PHC in Aboriginal Australia and was impressed by a 2018 scoping review conducted by Harfield *et al.* It was clear that the Indigenous PHC model proposed (Harfeild *et al.*) was consistent with the ideas put forward by the group members.

The collaborative approach taken by the Working Group is linked to the growing body of literature around opening up methods of working and learning together across the cultural divide (Coffin, 2007; Saha *et al.*, 2008; Tuhiwai-Smith, 2012; Horvat *et al.*, 2014; Bainbridge *et al.*, 2015). This collaborative, post-colonial approach can lead to the development of modes of service delivery that are inclusive of cultural differences and diversity.

Our authors include experienced Australian Aboriginal health professionals as well as colleagues and managers. These experienced health professionals offer an understanding of Indigenous beliefs and values from their community-based perspective. Their stories provide their professional observations. The first and last authors drafted the paper based on this collection of material which was then collaboratively reviewed by the authors.

## Discussion

Four of our authors are Australian Aboriginal health care professionals who have engaged in health care delivery in regional Western Australia for over two decades. We learned how they have built relationships with families in their care, yarning with family members and moderating competing priorities. This has enabled them to advocate on behalf of families in order to help them navigate the health care system.

Their stories form the basis of the recommendations of the Working Group, (Stroud *et al.*, 2020) emphasising the critical importance of deep Aboriginal involvement in the design and implementation of PHC in Aboriginal communities. Meetings of the Working Group focused on the central importance of Aboriginal leadership, and the notion of ‘empowerment’ was explored. The Working Group concluded that the key issues are more issues of enabling a local workforce, the importance of building capacity at a local level to create a sustainable workforce, and the provision of ongoing mentoring and support of those health professionals. This is a two-way process which also builds capacity of the non-Aboriginal workforce in their understanding of cultural issues and helps build team relationships.

Deep involvement of local Aboriginal people enables, reflects and integrates culture in its essence. It is about working together as a team as well as with the community.

### Learnings from community-based health professionals

If significant improvements are to be made in the health of Australian Aboriginal people in Australia, Aboriginal health professionals must play a key role in planning and leading health service delivery within their communities (Kildea *et al.*, 2016; Gomersall *et al.*, 2017; Middleton *et al.*, 2017; Jongen *et al.*, 2019; Marriott *et al.*, 2019).

While many Aboriginal Community-Controlled Health Organisations (ACCHOs) have been consistent in developing Aboriginal programme leadership, we have found this is not always the case for all health providers. Unfortunately, from personal experience, the majority of Aboriginal health professionals are not in senior positions within the health care system.

This experience is reiterated by Jongen *et al.* (Jongen *et al.*, 2019) who give a range of reasons why Aboriginal health professionals are not in senior positions, including limited career pathways, lack of support from management and lack of professional opportunities (Jongen *et al.*, 2019). This is also reflected in various community-led government documents and reports for ways of working (Aboriginal and Torres Strait Islander Health Workforce Working Group, 2017; Dawson *et al.*, 2020).

As a consequence, Aboriginal team members are seldom involved in the planning of health care programmes (Fogarty *et al.,* 2018a & 2018b). This is often related to a lack of cultural experience and knowledge on the part of non-Aboriginal staff, particularly those in management roles.

These messages and observations from across Western Australia are consistent with principles of the Indigenous PHC service delivery model proposed by Harfield *et al.* (2018) and reflected in ACCHOS ‘ways of working’ (Dawson *et al.*, 2020). These narratives provide useful examples of how Aboriginal health professionals can work effectively within a supportive and enabling team.

### Building future programmes

The impact of colonisation has had a profound influence on loss of traditional knowledge of health and wellbeing, ways of caring for country and loss of Indigenous languages (Malcolm, 1995). Other research demonstrates the ongoing strength of traditional knowledge, for example, ways of raising children (Lowell *et al.*, 2019). Acknowledging Aboriginal knowledge and incorporating learnings into service delivery systems will improve outcomes for health and wellbeing (Zubrick *et al.*, 2006; Coffin, 2007; Wright, 2011; Tuhiwai-Smith, 2012; Lowell *et al.*, 2019) and support language and learning skills in children (Ball & Lewis, 2014; Speech Pathology Australia 2019).

By integrating learnings that we have learned from our Aboriginal colleagues, we could make progress towards a collaborative service delivery approach. Such a model would integrate cultural values into its design and delivery, ultimately improving engagement with Aboriginal families.

### Limitations

We acknowledge that the opinions of our Aboriginal informants cannot be simply generalised.

## Conclusion

Primary prevention programmes for health and wellbeing in Aboriginal communities should be given greater importance if we are to successfully reduce the impact of diseases of childhood. We argue that prevention programmes require Aboriginal leadership and greater collaboration to ensure that cultural values and practices are embedded in the delivery of PHC. Most importantly, we argue for the inclusion of culture in PHC programmes, acknowledging local diversity, and stress the importance of Aboriginal leadership, integrating their wisdom, experience, and understanding of Aboriginal families and their communities.

Our findings from discussions with Aboriginal health professionals and their managers are consistent with the recommendations of Harfield *et al.* (2018). We concur with the recommendation of Harfield *et al.* (2018), that the Indigenous PHC model should be integrated into the design and implementation of collaborative approaches to building better PHC programmes with Australian Aboriginal communities.
